# A radiomics model based on preoperative gadoxetic acid–enhanced magnetic resonance imaging for predicting post-hepatectomy liver failure in patients with hepatocellular carcinoma

**DOI:** 10.3389/fonc.2023.1164739

**Published:** 2023-07-05

**Authors:** Changfeng Li, Qiang Wang, Mengda Zou, Ping Cai, Xuesong Li, Kai Feng, Leida Zhang, Ernesto Sparrelid, Torkel B. Brismar, Kuansheng Ma

**Affiliations:** ^1^ Institute of Hepatobiliary Surgery, Southwest Hospital, Army Medical University, Chongqing, China; ^2^ Division of Medical Imaging and Technology, Department of Clinical Science, Intervention and Technology (CLINTEC), Karolinska Institutet, Stockholm, Sweden; ^3^ Division of Radiology, Department of Clinical Science, Intervention and Technology (CLINTEC), Karolinska Institutet, Karolinska University Hospital, Stockholm, Sweden; ^4^ Department of Radiology, Southwest Hospital, Army Medical University, Chongqing, China; ^5^ Division of Surgery, Department of Clinical Science, Intervention and Technology (CLINTEC), Karolinska Institutet, Karolinska University Hospital, Stockholm, Sweden

**Keywords:** radiomics, magnetic resonance imaging, liver failure, hepatectomy, hepatocellular carcinoma

## Abstract

**Background:**

Post-hepatectomy liver failure (PHLF) is a fatal complication after liver resection in patients with hepatocellular carcinoma (HCC). It is of clinical importance to estimate the risk of PHLF preoperatively.

**Aims:**

This study aimed to develop and validate a prediction model based on preoperative gadoxetic acid–enhanced magnetic resonance imaging to estimate the risk of PHLF in patients with HCC.

**Methods:**

A total of 276 patients were retrospectively included and randomly divided into training and test cohorts (194:82). Clinicopathological variables were assessed to identify significant indicators for PHLF prediction. Radiomics features were extracted from the normal liver parenchyma at the hepatobiliary phase and the reproducible, robust and non-redundant ones were filtered for modeling. Prediction models were developed using clinicopathological variables (Clin-model), radiomics features (Rad-model), and their combination.

**Results:**

The PHLF incidence rate was 24% in the whole cohort. The combined model, consisting of albumin–bilirubin (ALBI) score, indocyanine green retention test at 15 min (ICG-R15), and Rad-score (derived from 16 radiomics features) outperformed the Clin-model and the Rad-model. It yielded an area under the receiver operating characteristic curve (AUC) of 0.84 (95% confidence interval (CI): 0.77–0.90) in the training cohort and 0.82 (95% CI: 0.72–0.91) in the test cohort. The model demonstrated a good consistency by the Hosmer–Lemeshow test and the calibration curve. The combined model was visualized as a nomogram for estimating individual risk of PHLF.

**Conclusion:**

A model combining clinicopathological risk factors and radiomics signature can be applied to identify patients with high risk of PHLF and serve as a decision aid when planning surgery treatment in patients with HCC.

## Introduction

Liver resection remains the mainstay modality in the treatment of hepatocellular carcinoma (HCC) with a curative intent. With the advances of surgical techniques and perioperative management in recent years, cases of extended or complex liver resection are increasing (1), which makes it increasingly important to make individual evaluations to avoid insufficient remnant liver volumes and impaired liver function after the surgery, the so-called post-hepatectomy liver failure (PHLF). At present, PHLF poses a fatal threat after liver resection and is the prominent cause of perioperative mortality (2), with a reported incidence as high as 40% (3).

Precise evaluation of liver function makes it possible to predict PHLF preoperatively. Previous studies have explored blood biochemistry tests, indocyanine green (ICG) test (4), and clinical scoring systems such as Child–Pugh score (5) and the Model for End-Stage Liver Disease (MELD) score (6) and computed tomography (CT)-based remnant liver volume (7) in the prediction of PHLF. However, the overall performance of these factors has been suboptimal. A more accurate, non-invasive approach for comprehensive liver function evaluation is urgently needed.

Gadoxetic acid (Primovist®) is a T1 magnetic resonance imaging (MRI) contrast medium widely used in clinical practice for liver lesion detection and characterization. Compared with the extracellular contrast media, it is actively taken up by hepatocyte at 10–40 min after administration (the so-called hepatobiliary phase) (8). Recent studies have shown that gadoxetic acid–enhanced MRI is promising in quantitative evaluation of liver function (9, 10). Classically, the methods used are based on the measurement of signal intensity (for instance relative liver enhancement or liver-to-muscle ratio or liver-to-spleen ratio), T1 relaxometry (e.g., T1 reduction rate), or dynamic contrast–enhanced MRI parameters (including hepatic extraction fraction) (11). These gadoxetic acid–enhanced MRI-derived parameters have shown a good correlation with ICG test and clinical scoring systems (Child–Pugh grades and MELD score), indicating a potential value in prediction of PHLF (10, 12, 13). However, when measuring signal intensity or T1 relaxation time, regions of interest (ROIs) with a limited diameter are most often placed in a single selected slice, which may not fully represent the whole liver function. Furthermore, the placement of the ROI is subjective, potentially reducing the reproducibility. In addition, the measurement of T1 relaxation time or dynamic contrast–enhanced MRI often requires additional scanning sequences (14).

Radiomics is a burgeoning technique, which can extract a great number of features from clinical routine medical imaging and transform them into mineable data for quantitative analysis (15). The basic assumption of radiomics is that the delicate pathophysiological alterations at cellular or molecular levels can be reflected by signal changes on images. The quantification of these imaging features and analyzing them through advanced algorithms or deep learning techniques can aid the clinician to solve clinical issues, such as disease diagnosis, prognosis, or prediction of treatment response. In the field of hepatobiliary imaging, previous studies have demonstrated that radiomics can significantly improve diagnostic and prognostic accuracy in HCC, such as the prediction of microvascular invasion (15), tumor differentiation (16), and early recurrence after hepatectomy (17).

In this study, it was tested whether radiomics analysis of gadoxetic-enhanced MR images can be used to predict PHLF in patients undergoing surgery due to HCC. The hypothesis was that radiomics analysis can detect delicate imaging features reflecting varying levels of liver function.

## Materials and methods

### Study design and patient selection

The research protocol of this single-center, retrospective study was reviewed and approved by the Institutional Review Board of Southwest Hospital, Army Medical University (No. (B)KY2021068). Written informed consent was waived due to the retrospective property of this study.

**Figure 1:**
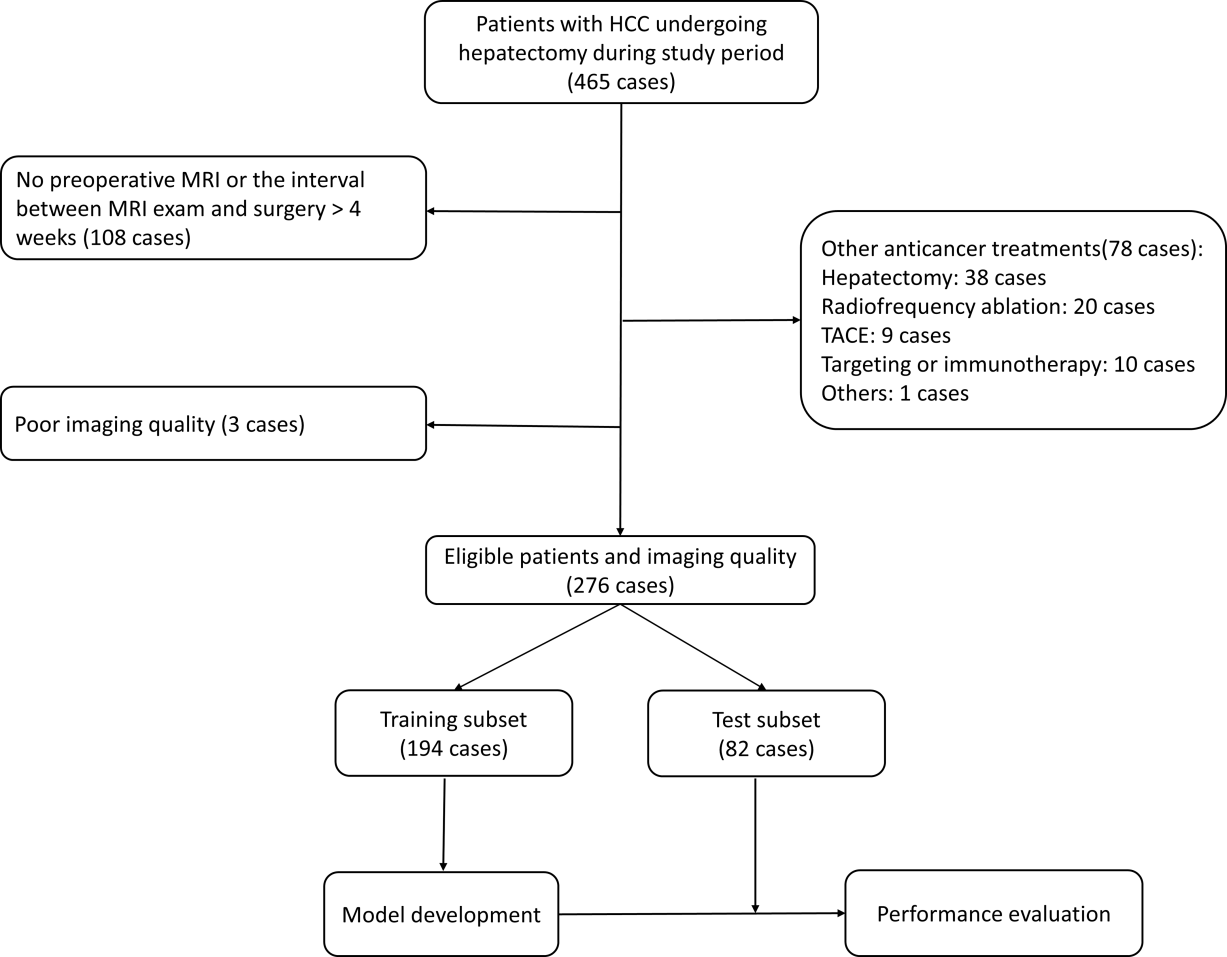
Flowchart of patient selection.

Consecutive patients undergoing hepatectomy during the period between January 2017 and March 2019 were retrieved according to the following inclusion and exclusion criteria. The inclusion criteria were 1) histopathologically confirmed HCC by resected specimen and 2) preoperative gadoxetic acid–enhanced MRI within 4 weeks before hepatectomy. The exclusion criteria were 1) anti-cancer treatment before hepatectomy, including radiofrequency ablation, hepatectomy, transarterial chemoembolization, portal vein embolization, targeted therapy, and immunotherapy, and 2) insufficient imaging quality (such as motion artifacts). In final, 276 patients were included in this study, and they were randomly divided into training and test cohorts at a ratio of 7:3, in which the training cohort was exclusively used for model development, while test cohort was used for to validate the performance of the model. **Figure 1** gives more details about this process.

**Figure 2:**
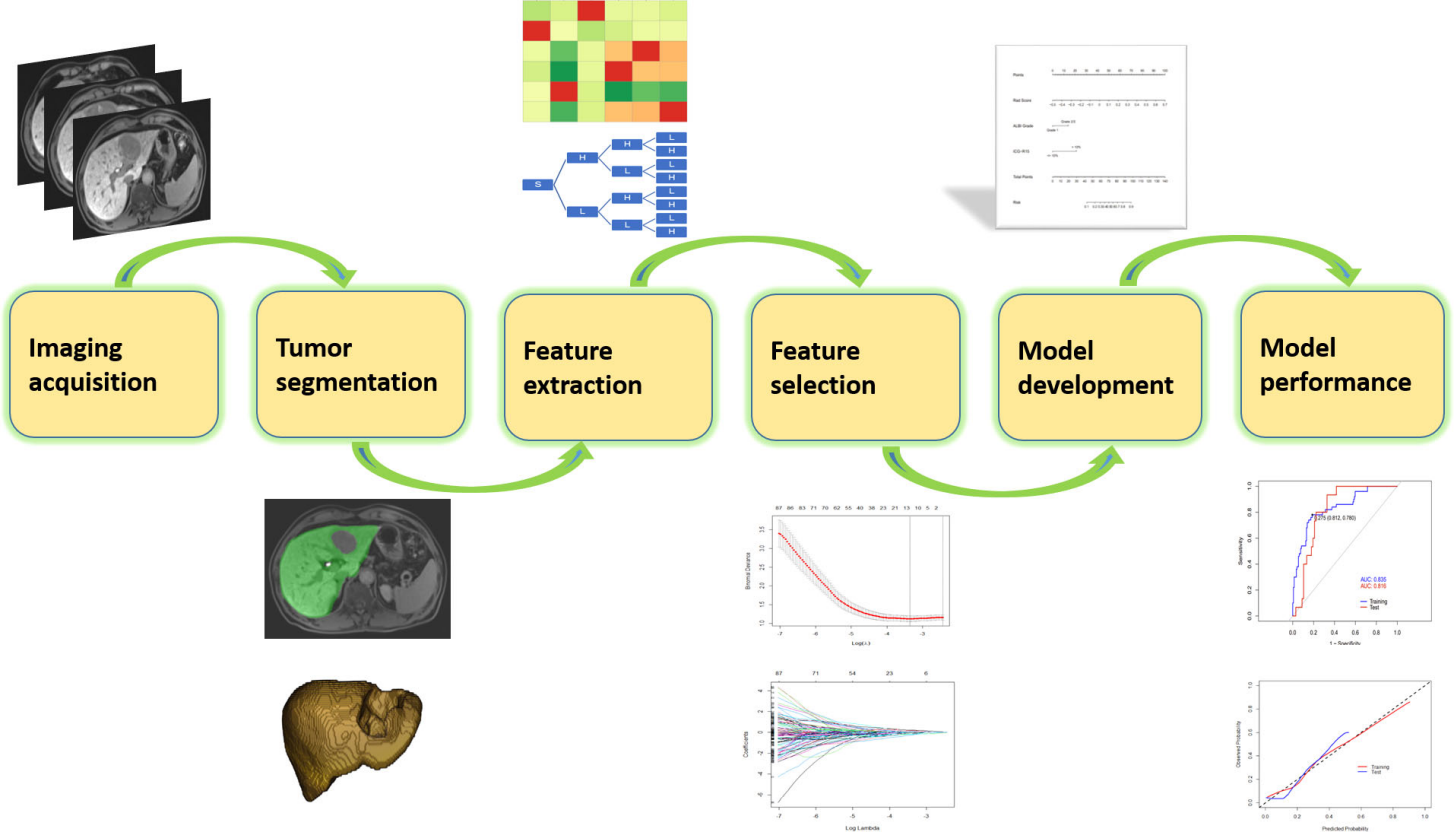
Workflow of the development of a radiomics-based model.

The reporting of this study followed the Checklist for Artificial Intelligence in Medical Imaging (CLAIM) guidance (18). The CLAIM checklist is provided at **Supplementary Table S1**. The process of model development is illustrated in **Figure 2**.

### Clinicopathological characteristics

Clinicopathological variables comprised of age, gender, body mass index, hepatitis B infection status, cirrhosis, tumor size, Child–Pugh grade, the albumin–bilirubin (ALBI) score (Grade 1 or Grade 2/3) (19), alanine aminotransferase (ALT), aspartate transaminase (AST), platelet, ICG-R15 test, liver resection extent (minor, if resected segments < 3 or major ≥ 3 segments) (20), laparoscopic-assisted operation, intraoperative blood loss, and liver resection duration. The scattered missing values were replaced by imputation of the median value.

### Definition of PHLF

PHLF was defined according to the International Study Group of Liver Surgery (ISGLS) standard: an increased international normalized ratio (INR) and hyperbilirubinemia (above the normal range of the local laboratory) on postoperative day 5 or afterwards (21). According to this definition, the patients were grouped into PHLF group and non-PHLF group.

### MR imaging acquisition

All patients underwent preoperative gadoxetic acid–enhanced MRI on a scanner (3.0 T, Magnetom Trio, Siemens Healthcare) with a six-channel body coil. Dynamic contrast–enhanced images were acquired using T1-weighted 3D volume interpolated breath hold sequence before, at the time of aorta enhancement, and 60 s, 180 s, 5 min, and 15 min after administration of contrast media. Gadoxetic acid (Primovist, Bayer Pharma, Berlin, Germany, 0.1 ml/kg body weight) was injected through an antecubital vein at a rate of 1.0 ml/s followed by a flush of saline at the same rate. Hepatobiliary phase was obtained at 15 min after injection. Detailed scanning parameters are provided in **Supplementary Table S2**.

### Delineation of normal liver tissue and inter-observer agreement evaluation

Delineation of normal liver tissue (exclusion of blood vessels, bile ducts, and cyst areas) was performed on images obtained in the hepatobiliary phase using the open-source software ITK-SNAP (http://www.itksnap.org/). Initially, 30 MR images were randomly selected for volume of interest (VOI) delineation by two researchers (with 2 years and 20 years of liver MRI experience, respectively) independently to evaluate reproducibility and stability of the extracted radiomics features. The inter-observer agreement was measured by interclass coefficient (ICC) on the VOI-based feature extraction. Features with ICC >0.75 were regarded as agreeable reproducibility and included for further analysis (22, 23). The liver delineation was then performed on the remaining patients by one researcher. The results were saved as a VOI file for further analysis. When contouring the liver, the researchers were blinded to the patients’ clinical information.

### Imaging preprocessing and radiomics feature extraction

Before feature extraction, all images were interpolated to a voxel size of 1 × 1 × 1 mm^3^, and the intensity histogram was discretized into a bin width of 25. A Python package, pyradiomics (https://github.com/AIM-Harvard/pyradiomics), was exploited to extract radiomics features from the manually delineated VOI. The terminology of the radiomics features extracted by pyradiomics is in accordance with the Image Biomarker Standardisation Initiative (24). The following categories of features were extracted: (1) shape, including 2D and 3D (n = 14); (2) first-order statistics (n = 18); (3) gray level co-occurrence matrix-derived feature (n = 24); (4) gray level run length matrix-derived feature (n = 16); (5) gray level size zone-derived feature (n = 16); (6) gray level dependence matrix-derived feature (n = 14); and (7) neighboring gray tone difference matrix feature (n = 5), (8) above features extracted from the wavelet transformed images (n = 744). In total, 851 features were exacted.

### Radiomics feature selection and radiomics model construction

In the training cohort, radiomics feature selection for the model construction involved two steps. First, after normalization of the radiomics features by *z*-score method, Spearman correlation analysis was performed among the features and only one of the pairs with a correlation coefficient >0.99 was kept in order to reduce redundancy. Second, the filtered features were fed into the least absolute shrinkage and selection operator (LASSO) regression analysis to detect the most informative features to avoid potential overfitting. The superparameter lambda (λ) in LASSO was determined by the fivefold cross-validation. Features with non-zero coefficient were selected for model development (termed as “Rad-model”).

### Clinical model construction

To detect independent risk factors for PHLF incidence, univariable regression analysis on clinicopathological variables were performed in the training cohort. The variables where the correlation to PHLF had a *p*-value <0.05 were used in a multivariable logistic regression analysis. A clinical model (coined as “Clin-model”) was then constructed using clinicopathological variables with *p* < 0.05 after the multivariable regression analysis.

### Combined model construction

Radiomics risk score (Rad-score) was then calculated for each patient through linear combination of included features in the Rad-model weighted by the corresponding coefficient. Clinicopathological variables in the Clin-model and the Rad-score were then collected to construct a combined model through logistic regression analysis. The ideal one was determined by the backward stepwise selection strategy using likelihood ratio test with Akaike information criteria (AIC) at the minimum value.

### Statistical analysis

Continuous variables with normal distribution were expressed as mean ± standard deviation and compared using Mann–Whitney U test between non-PHLF and PHLF groups. Categorical variables were presented as number (percentage) and were compared by Chi-square test or Fisher’s exact test. The performance of the models was evaluated based on their abilities of discrimination, calibration, and clinical usefulness in both training and test cohorts. The discrimination capability was assessed by the area under the receiver operating characteristic curve (AUC). Calibration capacity of the model was intuitively assessed by calibration curve. The goodness of fit of the model was measured by Hosmer–Lemeshow test, with *p*-value > 0.05 indicating a good result. Clinical usefulness of the model was evaluated by decision curve analysis (DCA). All statistical analyses were performed on R software (R Foundation for Statistical Computing, Vienna, Austria). A two-sided *p* < 0.05 was regarded as statistically significant.

## Results

### Patient basic characteristics

There were 238 men and 38 women among the 276 included patients, with a majority of patients <55 years (71.4%). According to the ISGLS criteria, 65 patients were diagnosed with PHLF, and the incidence rate of PHLF was 24% in the entire cohort. The training cohort contained 194 patients, and the test cohort contained 82 patients. The baseline characteristics between the two cohorts was balanced, with *p* > 0.05 for all variables, including the PHLF incidence. **Table 1** provides detailed information about the entire, training, and test cohorts.

**Table 1:** Patient clinicopathological characteristics.

Variables	Total (n=276)	Training cohort, No. (%)			Test cohort, No. (%)			*p*-value^a^
		PHLF (−) (n=144)	PHLF (+) (n=50)	*p*-value	PHLF (−) (n=67)	PHLF (+) (n=15)	*p*-value	
Gender				0.489			0.202	1.000
Female	38 (13.8)	22 (15.3)	5 (10.0)		11 (16.4)	0 (0.0)		
Male	238 (86.2)	122 (84.7)	45 (90.0)		56 (83.6)	15 (100.0)		
Age (years)				0.578			0.194	0.566
≤ 55	197 (71.4)	103 (71.5)	33 (66.0)		52 (77.6)	9 (60.0)		
> 55	79 (28.6)	41 (28.5)	17 (34.0)		15 (22.4)	6 (40.0)		
BMI (kg/m^2^)				0.274			0.334	1.000
≤ 18.5	6 (2.17)	2 (1.39)	2 (4.0)		1 (1.5)	1 (6.67)		
> 18.8	270 (97.8)	142 (98.6)	48 (96.0)		66 (98.5)	14 (93.3)		
Etiology of hepatitis				0.891			0.503	0.872
HBV	212 (76.8)	109 (75.7)	39 (78.0)		51 (76.1)	13 (86.7)		
None/others	64 (23.2)	35 (24.3)	11 (22.0)		16 (23.9)	2 (13.3)		
Cirrhosis status				1.000			0.641	0.817
Present	146 (52.9)	77 (53.5)	27 (54.0)		33 (49.3)	9 (60.0)		
Absent	130 (47.1)	67 (46.5)	23 (46.0)		34 (50.7)	6 (40.0)		
ALT (IU/L)				0.220			0.087	0.147
≤ 42	158 (57.2)	91 (63.2)	26 (52.0)		37 (55.2)	4 (26.7)		
> 42	118 (42.8)	53 (36.8)	24 (48.0)		30 (44.8)	11 (73.3)		
AST (IU/L)				0.018			0.955	1.000
≤ 42	158 (57.2)	90 (62.5)	21 (42.0)		39 (58.2)	8 (53.3)		
> 42	118 (42.8)	54 (37.5)	29 (58.0)		28 (41.8)	7 (46.7)		
Platelet (×10^9^/L)				0.013			0.475	1.000
≤ 125	96 (34.8)	42 (29.2)	25 (50.0)		22 (32.8)	7 (46.7)		
> 125	180 (65.2)	102 (70.8)	25 (50.0)		45 (67.2)	8 (53.3)		
Child–Pugh grade				1.000			0.183	1.000
A	272 (98.6)	142 (98.6)	49 (98.0)		67 (100.0)	14 (93.3)		
B	4 (1.45)	2 (1.39)	1 (2.0)		0 (0.0)	1 (6.7)		
ALBI score				0.001			0.003	0.282
Grade 1	126 (45.7)	73 (50.7)	11 (22.0)		40 (59.7)	2 (13.3)		
Grade 2/3	150 (54.3)	71 (49.3)	39 (78.0)		27 (40.3)	13 (86.7)		
ICG-R15 test				<0.001			1.000	0.986
≤ 10%	254 (92.0)	139 (96.5)	39 (78.0)		62 (92.5)	14 (93.3)		
> 10%	22 (7.97)	5 (3.47)	11 (22.0)		5 (7.46)	1 (6.67)		
Tumor size (cm)				0.739			0.602	0.410
≤ 5	130 (47.1)	69 (47.9)	26 (52.0)		30 (44.8)	5 (33.3)		
> 5	146 (52.9)	75 (52.1)	24 (48.0)		37 (55.2)	10 (66.7)		
Resection extent				0.629			1.000	0.832
Minor	196 (71.0)	105 (72.9)	34 (68.0)		46 (68.7)	11 (73.3)		
Major	80 (29.0)	39 (27.1)	16 (32.0)		21 (31.3)	4 (26.7)		
Laparoscopic operation				1.000			0.218	1.000
Yes	42 (15.2)	22 (15.3)	8 (16.0)		8 (11.9)	4 (26.7)		
No	234 (84.8)	122 (84.7)	42 (84.0)		59 (88.1)	11 (73.3)		
Intraoperative blood loss (ml)				0.553			0.459	0.857
≤ 400	222 (80.4)	117 (81.2)	38 (76.0)		56 (83.6)	11 (73.3)		
> 400	54 (19.6)	27 (18.8)	12 (24.0)		11 (16.4)	4 (26.7)		
Liver resection time (min)				0.549			1.000	0.071
≤ 60	227 (82.3)	125 (86.8)	41 (82.0)		50 (75.8)	12 (75.0)		
> 60	49 (17.7)	19 (13.2)	9 (18.0)		16 (24.2)	4 (25.0)		
MELD score				0.744			0.151	0.763
≤ 9	259 (93.8)	135 (93.8)	46 (92.0)		65 (97.0)	13 (86.7)		
> 9	17 (6.16)	9 (6.25)	4 (8.0)		2 (3.0)	2 (13.3)		

ALBI score, albumin–bilirubin score; ALT, alanine transaminase; AST, aspartate transaminase; BMI, body mass index; HBV, hepatitis B virus; ICG-R15, indocyanine green retention rate at 15 min; MELD score, Model for End-Stage Liver Disease score; PHLF, post-hepatectomy liver failure.

a Between training and test cohorts. Data are expressed as n (%).

### Clinical model construction

Based on univariable and multivariable logistic regression analyses, three significant clinicopathological variables were detected, including platelet, ALBI score, and ICG-R15 (*p* < 0.05) (**Table 2**). The Clin-model was based on these three variables. The AUC of the Clin-model in the training and the test cohort was 0.74 (95% confidence interval, CI: 0.65–0.83) and 0.71 (95% CI: 0.57–0.84) respectively (**Table 3**). The formula of the Clin-model is provided in **Supplementary Material S1**.

**Table 2:** Clinicopathological risk factors for post-hepatectomy liver failure in patients with hepatocellular carcinoma.

Variables	Univariable analysis		Multivariable analysis	
	OR (95% CI)	*p*-value	OR (95% CI)	*p*-value
Gender				
Male vs. female	1.62 (0.58–4.54)	0.357		
Age (years)				
> 55 vs. ≤ 55	1.29 (0.65–2.58)	0.463		
BMI (kg/m^2^)				
≤ 18.5 vs. > 18.5	2.96 (0.35–25.21)	0.285		
Etiology of hepatitis				
HBV vs. none/others	1.14 (0.53–2.46)	0.741		
Cirrhosis status				
Present vs. absent	1.02 (0.54–1.95)	0.949		
ALT (IU/L)				
> 42 vs. ≤ 42	1.59 (0.83–3.04)	0.165		
AST (IU/L)				
> 42 vs. ≤ 42	2.30 (1.20–4.43)	0.013	1.69 (0.82–3.48)	0.153
Platelet (×109/L)				
≤ 125 vs. > 125	2.43 (1.26–4.73)	0.008	2.50 (1.22–5.17)	0.013
Child–Pugh grade				
B vs. A	1.45 (0.13–16.33)	0.764		
ALBI score				
Grade 2/3 vs. Grade 1	3.65 (1.73–7.68)	0.001	3.20 (1.46–7.00)	0.004
ICG-R15 test				
> 10% vs. ≤ 10%	7.84 (2.57–23.92)	<0.001	4.87 (1.5–16.02)	0.009
Tumor size (cm)				
≤ 5 vs. > 5	1.18 (0.62–2.25)	0.619		
Resection extent				
Major vs. minor	1.27 (0.63–2.55)	0.507		
Laparoscopic operation				
Yes vs. no	1.06 (0.42–2.47)	0.903		
Intraoperative blood loss (ml)				
> 400 vs. ≤ 400	1.37 (0.63–2.96)	0.421		
Liver resection time (min)				
> 60 vs. ≤ 60	1.44 (0.61–3.44)	0.407		
MELD score				
> 9 vs. ≤ 9	1.30 (0.38–4.44)	0.671		

ALBI score, albumin–bilirubin score; ALT, alanine transaminase; AST, aspartate transaminase; BMI, body mass index; CI, confidence interval; HBV, hepatitis B virus; ICG-R15, indocyanine green retention rate at 15 min; MELD score, Model for End-Stage Liver Disease score; OR, odds ratio.

**Table 3:** Performance of the models for post-hepatectomy liver failure prediction in training and test cohorts.

		Clin-model	Rad-model	Combined model
Training cohort	Cut-off value	0.27	0.29	0.28
	AUC (95% CI)	0.74 (0.65–0.83)	0.79 (0.72–0.86)	0.84 (0.77–0.90)
	Sensitivity	0.70	0.70	0.78
	Specificity	0.74	0.76	0.81
	PPV	0.49	0.51	0.59
	NPV	0.88	0.88	0.91
	Accuracy	0.73	0.75	0.80
Test cohort	AUC (95% CI)	0.71 (0.57–0.84)	0.79 (0.69–0.89)	0.82 (0.72–0.91)
	Sensitivity	0.87	0.87	0.93
	Specificity	0.55	0.70	0.67
	PPV	0.30	0.39	0.39
	NPV	0.95	0.96	0.98
	Accuracy	0.61	0.73	0.72

AUC, area under the receiver operating characteristics curve; CI, confidence interval; PPV, positive predictive value; NPV, negative predictive value.

### Radiomics feature selection and model construction

**Figure 3:**
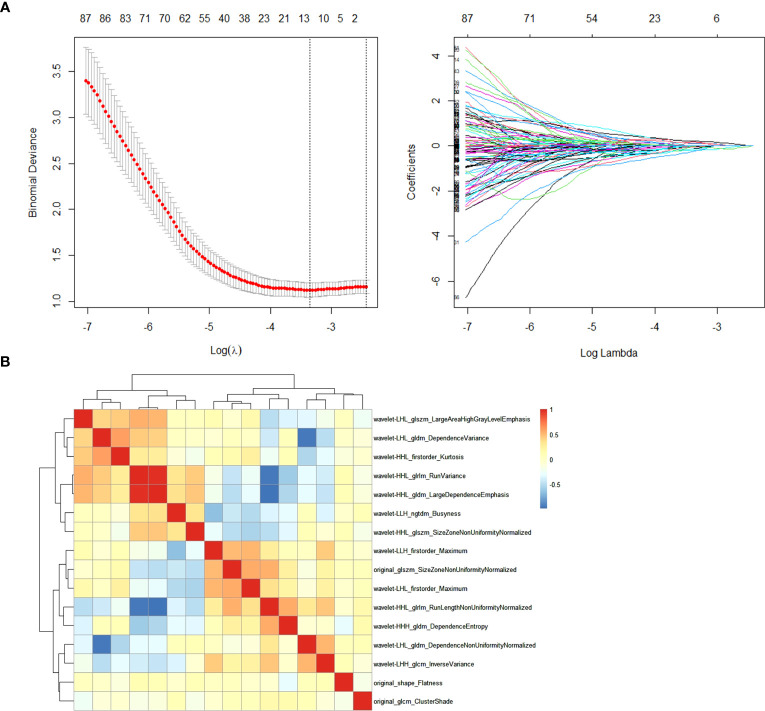
**(A)** Feature selection through the least absolute shrinkage and selection operator (LASSO) algorithm. **(B)** Heatmap of the correlation coefficient matrix of the selected 16 features through LASSO.

Among the 851 extracted radiomics features, 494 features (58%) showed an ICC ≥ 0.75, and these features were subjected to the two-step feature selection strategy. In the first step, 315 features remained after excluding one of paired features with a correlation coefficient > 0.99. In the second step, 16 non-zero coefficient features were selected by LASSO-logistic regression analysis (**Figure 3**) and were subsequently used for constructing the Rad-model. The Rad-model had an AUC of 0.79 (95% CI: 0.72–0.86) in the training cohort and 0.79 (95% CI: 0.69–0.89) in the test cohort (**Table 3**). The difference in the Clin-model and the Rad-model in performance was not significant (Delong test, *p* = 0.24).

### Combined model construction

The individual Rad-score was calculated through a linear combination of the included variables in the Rad-model weighted by the corresponding coefficients (**Supplementary Figure S1** and **Supplementary Material S2**). A third model, the combined model, was then constructed according to the AIC minimum value, which includes ALBI score, ICG-R15, and rad-score.

### Performance evaluation of the combined model

**Figure 4:**
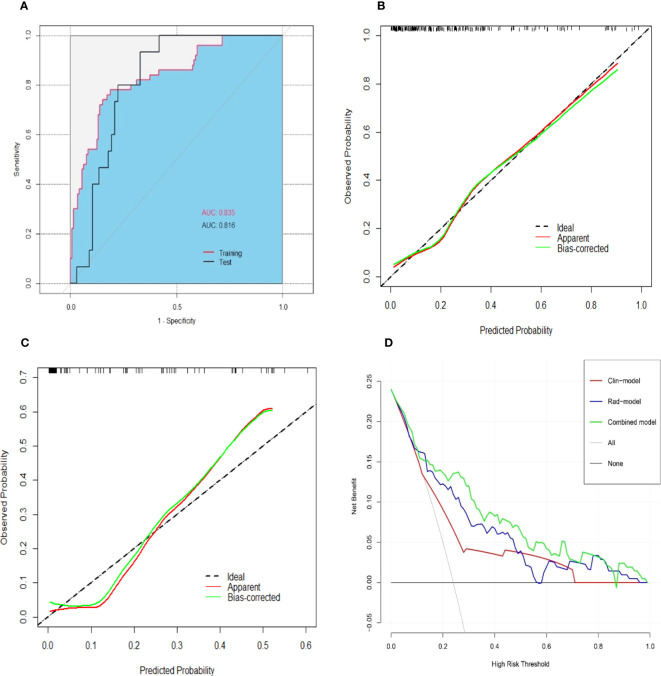
Performance of the combined model for predicting post-hepatectomy liver failure. An area under the receiver operating characteristic curve in the training and test cohorts **(A)**. Calibration curves in the training **(B)** and test cohorts **(C)** illustrated a good consistency between the model-predicted probability and the actual probability of PHLF. The red line stands for the combined model, while the green line describes the combined model calibrated by 1,000 bootstrap resampling strategy. The dash line indicates an ideal situation that the model-predicted probability perfectly matches the actual probability of PHLF. The decision curve analysis **(D)** showed that the combined model (green line) yielded a highest net benefit at different risk threshold of PHLF, compared with the clinical model (red line) and the radiomics model (blue line). Note: AUC, area under the receiver operating characteristic curve; PHLF, post-hepatectomy liver failure.

**Figure 5:**
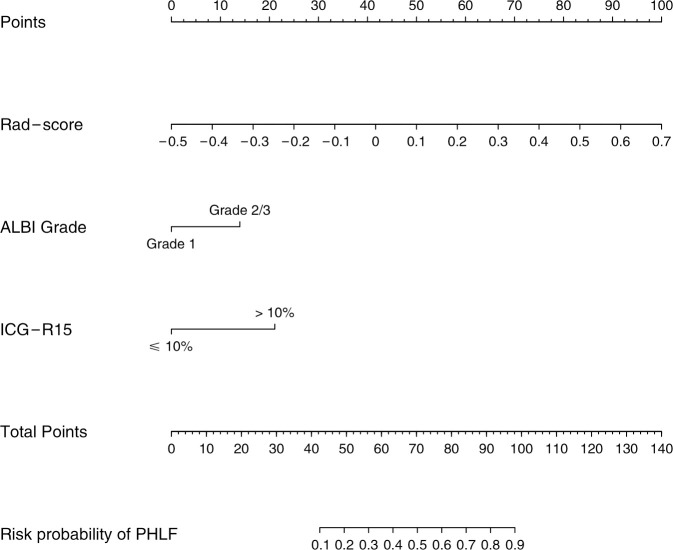
Nomogram for predicting post-hepatectomy liver failure in patients with hepatocellular carcinoma. ALBI, the albumin–bilirubin score; ICG-R15, indocyanine green retention rate at 15 min; PHLF, post-hepatectomy liver failure.

The combined model yielded an AUC of 0.84 (95% CI: 0.77–0.90) in the training cohort, with sensitivity of 0.78 and specificity of 0.81 (**Figure 4A**). It exhibited an AUC of 0.82 (95% CI: 0.72–0.91) and sensitivity of 0.93 and 0.67 in the test cohort (**Table 3**). The AUC difference was significant between the combined model and the Clin-model (*p* < 0.05), but not between the combined model and the Rad-model (*p* = 0.08, **Table 3**). The combined model has been visualized as a nomogram (**Figure 5**) for clinical utility. An online tool to facilitate its calculation is available at https://onlinetools.shinyapps.io/onlineTool/.

The optimal cutoff value of the model was set at 0.28. The calibration curve showed a good agreement between the combined model predicted values and observed PHLF rate (Hosmer–Lemeshow test *p* > 0.05) (**Figures 4B, C**). The DCA plot illustrated that compared with the “treat-all” and “treat-none” strategies, the net benefit was higher for the combined model than the Clin-model and the Rad-model, implying that the combined model was beneficial for clinical utility (**Figure 4D**).

## Discussion

To predict PHLF in patients with HCC, we developed and validated a nomogram model combining two clinicopathological variables (ALBI score and ICG-R15) and one radiomics variable (Rad-score) derived from radiomics analysis of preoperative T1-weighted gadoxetic acid–enhanced MRI. This prediction model yielded an AUC of 0.82 in the test cohort, indicating a promising tool for clinical utility.

Until now, only few studies have explored radiomics for prediction of PHLF. Zhu et al. proposed a nomogram model including ICG-R15 and radiomics signature based on hepatobiliary phase of gadoxetic acid–enhanced MRI from 101 patients (25). The model yielded an AUC of 0.89 in prediction PHLF in patients undergoing major liver resection. However, the study did not further validate the model in an independent test cohort. Chen et al. developed a combined model incorporating platelet, tumor size, and radiomics score deriving from preoperative gadoxetic acid–enhanced MRI for predicting PHLF (26). They validated the model at another medical center, obtaining an AUC of 0.84. However, in that study, they did not present their model with a formula or nomogram, which made it hard to reproduce or translate their model into clinical utility. In addition, their radiomics analysis was based on a single MRI slice per patient, which may not fully reflect the liver function. There are also two radiomics models based on CT modality for PHLF prediction (27, 28). Those studies had a rather limited sample size (112 and 186 cases), which may explain the unusual outcome with AUCs in their respective test cohorts higher than that in their training cohorts (27, 28).

The Rad-model alone showed an effective prediction efficacy, almost comparable to our combined model. A majority of the radiomics features in the Rad-model (13/16) belonged to wavelet-derived features. Those described low and high frequency signals, representing homogeneity and heterogeneity of the liver tissue (29). Unfortunately, the two previously published studies on radiomics of hepatobiliary phase of gadoxetic acid–enhanced MRI, by Zhu and Chen as mentioned above, did not adopt wavelet filter, so it is not possible to make comparisons regarding the specific radiomics features. However, wavelet-derived features do frequently appear in other gadoxetic acid–enhanced MRI radiomics models, for instance, in the prediction of microvascular invasion (30) or tumor grading for HCC (31), indicating that they capture important structural features of hepatic tumor or parenchyma (14).

Another variable in our prediction model is ICG-R15. This was consistent with Zhu’s PHLF prediction model, in which ICG-R15 was the only clinical predictor (25). Currently, ICG-R15 still serves as a reference standard in the quantitative evaluation of liver function before liver resection and plays an essential role in treatment management of HCC patients (32, 33). Nevertheless, the role of ICG test as an independent risk factor for PHLF prediction remains controversial, as it can be influenced by many factors, such as blood flow or hyperbilirubinemia (32, 34). This might explain why only approximately half of currently available studies (5/11) could successfully use ICG to predict PHLF as shown in a systematnic review (3).

Our model also consists of a predictor of ALBI grade, which is a simple and objective scoring system adopting just two common biochemistry tests (serum albumin and bilirubin) for quantitative evaluation of liver function in HCC patients (19). It was proposed to overcome the limitation of the conventional Child–Pugh scoring system and has proven to be a reliable, effective tool for liver function evaluation, applicable in several different geographic regions (19). Xiang et al. have shown that ALBI could predict PHLF with an AUC of 0.64 in the test cohort (28). In the multivariable regression analysis for Clin-model, ALBI grade demonstrated an independent risk factor for PHLF incidence (odds ratio: 3.2, Grade 2/3 vs. Grade 1). However, neither Child–Pugh nor MELD score was a significant risk factor for PHLF prediction in our cohort.

This research has some limitations to be acknowledged. First, the retrospective nature of this study bore incoherent selection biases that could have had an impact on the results. However, this issue was partially compensated *via* inclusion of consecutive patients. Second, our model was not validated in an external cohort. Additional validation in larger prospective multicenter cohorts is warranted to generalize our prediction model. Third, the radiomics analysis was performed based on the whole normal liver parenchyma, rather than the future liver remnant (FLR) only. A study based on FLR only might show better AUC than presented here. The large AUC observed in our study might be explained by a strong relationship in radiomics between FLR and resected part. Lastly, the resection extent was not included in our prediction model, as it was not significant during univariable logistic regression analysis. Traditionally, the hepatic resection extent is regarded as an important indicator for PHLF. However, its role may be impaired with the development of surgical concepts and skills, equipment, perioperative management, and anesthesia techniques. Currently, the occurrence of PHLF is assumed as a consequence of multiple clinicopathological factors during the perioperative period, including baseline liver/patient characteristics and intraoperative and postoperative factors (35). Interestingly, among the four existing studies that developed the radiomics models for PHLF prediction using preoperative imaging (25–28), only one study detected the resection extent significant and included it in their model (28). Furthermore, we compared the difference in the prediction performance between our proposed models with and without the variable of the resection extent, and the test showed an insignificant result (**Supplementary Table S3**). Due to the simplicity principle, this variable was not included in our final models. Future studies can further investigate the effect of this variable on the incidence of PHLF.

In conclusion, a prediction nomogram combining clinical risk factors and radiomics signature based on preoperative gadoxetic acid–enhanced MRI was constructed, and it can potentially be an effective tool for predicting liver failure after liver resection in patients with hepatocellular carcinoma.

## Data availability statement

The original contributions presented in the study are included in the article/**Supplementary Material**. Further inquiries can be directed to the corresponding authors.

## Ethics statement

The studies involving human participants were reviewed and approved by The Institutional Review Board of Southwest Hospital, Army Medical University. The ethics committee waived the requirement of written informed consent for participation.

## Author contributions

Conceptualization: KM and QW. Data curation: CL, QW, PC, MZ, XL, KF, and LZ. Formal analysis: CL, QW, and KM. Funding acquisition: KM and QW. Investigation: CL, MZ, XL, and KF. Methodology: KM, QW, and TB. Project administration: KM and LZ. Software: PC and XL. Supervision: KM, TB, LZ, and ES. Writing—original draft: CL and QW. Writing—review and editing: all authors. All authors contributed to the article and approved the submitted version.

## References

[1] Jarnagin WR Gonen M Fong Y DeMatteo RP Ben-Porat L Little S. Improvement in perioperative outcome after hepatic resection: analysis of 1,803 consecutive cases over the past decade. Ann Surg (2002) 236:397–407. doi: 10.1097/01.SLA.0000029003.66466.B3 12368667 PMC1422593

[2] Rassam F Zhang T Cieslak KP Lavini C Stoker J Bennink RJ. Comparison between dynamic gadoxetate-enhanced MRI and ^99m^Tc-mebrofenin hepatobiliary scintigraphy with SPECT for quantitative assessment of liver function. Eur Radiol (2019) 29:5063–72. doi: 10.1007/s00330-019-06029-7 PMC6682576 30796575

[3] Wang Q Wang A Sparrelid E Zhang J Zhao Y Ma K. Predictive value of gadoxetic acid-enhanced MRI for posthepatectomy liver failure: a systematic review. Eur Radiol (2022) 32:1792–803. doi: 10.1007/s00330-021-08297-8 PMC8831250 34562137

[4] Sunagawa Y Yamada S Kato Y Sonohara F Takami H Inokawa Y. Perioperative assessment of indocyanine green elimination rate accurately predicts postoperative liver failure in patients undergoing hepatectomy. J Hepatobiliary Pancreat Sci (2021) 28:86–94. doi: 10.1002/jhbp.833 33052632

[5] Zou H Yang X Li QL Zhou QX Xiong L Wen Y. A comparative study of albumin-bilirubin score with child-pugh score, model for end-stage liver disease score and indocyanine green R15 in predicting posthepatectomy liver failure for hepatocellular carcinoma patients. Dig Dis (2018) 36:236–43. doi: 10.1159/000486590 29495004

[6] Chin KM Allen JC Teo JY Kam JH Tan EK Koh Y. Predictors of post-hepatectomy liver failure in patients undergoing extensive liver resections for hepatocellular carcinoma. Ann Hepatobiliary Pancreat Surg (2018) 22:185–96. doi: 10.14701/ahbps.2018.22.3.185 PMC6125273 30215040

[7] Yamamoto R Sugiura T Okamura Y Ito T Yamamoto Y Ashida R. Utility of remnant liver volume for predicting posthepatectomy liver failure after hepatectomy with extrahepatic bile duct resection. BJS Open (2021) 5:zraa049. doi: 10.1093/bjsopen/zraa049 33609394 PMC7893452

[8] Brismar TB Dahlstrom N Edsborg N Persson A Smedby O Albiin N. Liver vessel enhancement by gd-BOPTA and gd-EOB-DTPA: a comparison in healthy volunteers. Acta Radiol Sep (2009) 50:709–15. doi: 10.1080/02841850903055603 19701821

[9] Bastati N Beer L Mandorfer M Poetter-Lang S Tamandl D Bican Y. Does the functional liver imaging score derived from gadoxetic acid-enhanced MRI predict outcomes in chronic liver disease? Radiology. (2020) 294:98–107. doi: 10.1148/radiol.2019190734 31743083

[10] Yoon JH Lee JM Kang HJ Ahn SJ Yang H Kim E. Quantitative assessment of liver function by using gadoxetic acid-enhanced MRI: hepatocyte uptake ratio. Radiology. (2019) 290:125–33. doi: 10.1148/radiol.2018180753 30375932

[11] Bae KE Kim SY Lee SS Kim KW Won HJ Shin YM. Assessment of hepatic function with gd-EOB-DTPA-enhanced hepatic MRI. Dig Dis (2012) 30:617–22. doi: 10.1159/000343092 23258104

[12] Nilsson H Blomqvist L Douglas L Nordell A Janczewska I Näslund E. Gd-EOB-DTPA-enhanced MRI for the assessment of liver function and volume in liver cirrhosis. Br J Radiol (2013) 86:20120653. doi: 10.1259/bjr.20120653 23403453 PMC3664988

[13] Kim YC Kim MJ Park YN Kim KS Ahn SH Jung SE. Relationship between severity of liver dysfunction and the relative ratio of liver to aortic enhancement (RE) on MRI using hepatocyte-specific contrast. J Magn Reson Imaging (2014) 39:24–30. doi: 10.1002/jmri.24100 23553935

[14] Lambin P Leijenaar RTH Deist TM Peerlings J de Jong EEC van Timmeren J. Radiomics: the bridge between medical imaging and personalized medicine. Nat Rev Clin Oncol (2017) 14:749–62. doi: 10.1038/nrclinonc.2017.141 28975929

[15] Wang Q Li C Zhang J Hu X Fan Y Ma K. Radiomics models for predicting microvascular invasion in hepatocellular carcinoma: a systematic review and radiomics quality score assessment. Cancers (Basel) (2021) 13:5864. doi: 10.3390/cancers13225864 34831018 PMC8616379

[16] Ding Y Ruan S Wang Y Shao J Sun R Tian W. Novel deep learning radiomics model for preoperative evaluation of hepatocellular carcinoma differentiation based on computed tomography data. Clin Transl Med (2021) 11:e570. doi: 10.1002/ctm2.570 34841694 PMC8571950

[17] Zhao Y Wu J Zhang Q Hua Z Qi W Wang N. Radiomics analysis based on multiparametric MRI for predicting early recurrence in hepatocellular carcinoma after partial hepatectomy. J Magn Reson Imaging (2021) 53:1066–79. doi: 10.1002/jmri.27424 33217114

[18] Mongan J Moy L Kahn CE Jr. Checklist for artificial intelligence in medical imaging (CLAIM): a guide for authors and reviewers. Radiol Artif Intell (2020) 2:e200029. doi: 10.1148/ryai.2020200029 33937821 PMC8017414

[19] Johnson PJ Berhane S Kagebayashi C Satomura S Teng M Reeves HL. Assessment of liver function in patients with hepatocellular carcinoma: a new evidence-based approach-the ALBI grade. J Clin Oncol (2015) 33:550–8. doi: 10.1200/JCO.2014.57.9151 PMC4322258 25512453

[20] Dahiya D Wu TJ Lee CF Chan KM Lee WC Chen MF. Minor versus major hepatic resection for small hepatocellular carcinoma (HCC) in cirrhotic patients: a 20-year experience. Surgery. (2010) 147:676–85. doi: 10.1016/j.surg.2009.10.043 20004441

[21] Rahbari NN Garden OJ Padbury R Brooke-Smith M Crawford M Adam R. Posthepatectomy liver failure: a definition and grading by the international study group of liver surgery (ISGLS). Surgery. (2011) 149:713–24. doi: 10.1016/j.surg.2010.10.001 21236455

[22] Koo TK Li MY. A guideline of selecting and reporting intraclass correlation coefficients for reliability research. J Chiropr Med (2016) 15:155–63. doi: 10.1016/j.jcm.2016.02.012 PMC4913118 27330520

[23] Gu D Xie Y Wei J Li W Ye Z Zhu Z. MRI-Based radiomics signature: a potential biomarker for identifying glypican 3-positive hepatocellular carcinoma. J Magn Reson Imaging (2020) 52:1679–87. doi: 10.1002/jmri.27199 32491239

[24] Zwanenburg A Vallières M Abdalah MA Aerts HJWL Andrearczyk V Apte A. The image biomarker standardization initiative: standardized quantitative radiomics for high-throughput image-based phenotyping. Radiology. (2020) 295:328–38. doi: 10.1148/radiol.2020191145 PMC7193906 32154773

[25] Zhu WS Shi SY Yang ZH Song C Shen J. Radiomics model based on preoperative gadoxetic acid-enhanced MRI for predicting liver failure. World J Gastroenterol (2020) 26:1208–20. doi: 10.3748/wjg.v26.i11.1208 PMC7093309 32231424

[26] Chen Y Liu Z Mo Y Li B Zhou Q Peng S. Prediction of post-hepatectomy liver failure in patients with hepatocellular carcinoma based on radiomics using gd-EOB-DTPA-Enhanced MRI: the liver failure model. Front Oncol (2021) 11:605296. doi: 10.3389/fonc.2021.605296 33777748 PMC7987905

[27] Cai W He B Hu M Zhang W Xiao D Yu H. A radiomics-based nomogram for the preoperative prediction of posthepatectomy liver failure in patients with hepatocellular carcinoma. Surg Oncol (2019) 28:78–85. doi: 10.1016/j.suronc.2018.11.013 30851917

[28] Xiang F Liang X Yang L Liu X Yan S. CT radiomics nomogram for the preoperative prediction of severe post-hepatectomy liver failure in patients with huge (≥ 10 cm) hepatocellular carcinoma. World J Surg Oncol (2021) 19:344. doi: 10.1186/s12957-021-02459-0 34895260 PMC8667454

[29] Zhou J Lu J Gao C Zeng J Zhou C Lai X. Predicting the response to neoadjuvant chemotherapy for breast cancer: wavelet transforming radiomics in MRI. BMC Canc (2020) 20:100. doi: 10.1186/s12885-020-6523-2 PMC7003343 32024483

[30] Xu X Zhang HL Liu QP Sun SW Zhang J Zhu FP. Radiomic analysis of contrast-enhanced CT predicts microvascular invasion and outcome in hepatocellular carcinoma. J Hepatol (2019) 70:1133–44. doi: 10.1016/j.jhep.2019.02.023 30876945

[31] Wu M Tan H Gao F Hai J Ning P Chen J. Predicting the grade of hepatocellular carcinoma based on non-contrast-enhanced MRI radiomics signature. Eur Radiol (2019) 29:2802–11. doi: 10.1007/s00330-018-5787-2 30406313

[32] Vos JJ Wietasch JK Absalom AR Hendriks HG Scheeren TW. Green light for liver function monitoring using indocyanine green? an overview of current clinical applications. Anaesthesia. (2014) 69:1364–76. doi: 10.1111/anae.12755 24894115

[33] Clavien PA Petrowsky H DeOliveira ML Graf R. Strategies for safer liver surgery and partial liver transplantation. N Engl J Med (2007) 356:1545–59. doi: 10.1056/NEJMra065156 17429086

[34] Iimuro Y. ICG clearance test and 99mTc-GSA SPECT/CT fusion images. Visc Med (2017) 33:449–54. doi: 10.1159/000479046 PMC5757532 29344519

[35] Shen YN Zheng ML Guo CX Bai XL Pan Y Yao WY. The role of imaging in prediction of post-hepatectomy liver failure. Clin Imag (2018) 52:137–45. doi: 10.1016/j.clinimag.2018.07.019 30059953

