# Supplementary material for: A radiomics model based on preoperative gadoxetic acid–enhanced magnetic resonance imaging for predicting post-hepatectomy liver failure in patients with hepatocellular carcinoma
Source: Front Oncol. 2023 Jul 5;13:1164739. doi: 10.3389/fonc.2023.1164739 (PMC10354521; doi:10.3389/fonc.2023.1164739)
Supplement: Supplementary file 1 [file DataSheet_1.docx]

**Supplementary Material**

**A radiomics model based on preoperative gadoxetic acid enhanced magnetic resonance imaging for predicting posthepatectomy liver failure in patients with hepatocellular carcinoma**

Changfeng Li, Qiang Wang*, Mengda Zou, Ping Cai, Xuesong Li, Kai Feng, Leida Zhang, Ernesto Sparrelid, Torkel B. Brismar, Kuansheng Ma*

Supplementary Table S1. CLAIM checklist.

Supplementary Table S2. Gadoxetic acid enhanced MRI scanning parameters.

Supplementary Material S1. Formula of the clinical model (Clin-model).

Supplementary Material S2. Formula for calculation of Rad-score based on Rad-model.

Supplementary Figure S1. Rad-score in training and test cohorts.

Supplementary Table S3. Comparison between the models with and without a variable of “hepatic resection extent”.

Supplementary Table S1. CLAIM checklist.

| **No.** | **Item** | **Section / Topic** |
| --- | --- | --- |
|  |  | **TITLE / ABSTRACT** |
| 1 | Identification as a study of AI methodology, specifying the category of technology used (e.g., deep learning) | Page 1 |
| 2 | Structured summary of study design, methods, results, and conclusions | Page 1 |
|  |  | **INTRODUCTION** |
| 3 | Scientific and clinical background, including the intended use and clinical role of the AI approach | Page 2 |
| 4 | Study objectives and hypotheses | Page 2 |
|  |  | **METHODS** |
| 5 | Prospective or retrospective study | Page 2-3 |
| 6 | Study goal, such as model creation, exploratory study, feasibility study, non-inferiority trial | Page 2 |
| 7 | Data sources | Page 2-3 |
| 8 | Eligibility criteria: how, where, and when potentially eligible participants or studies were identified (e.g., symptoms, results from previous tests, inclusion in registry, patient-care setting, location, dates) | Page 2-3 |
| 9 | Data pre-processing steps | Page 3-4 |
| 10 | Selection of data subsets, if applicable | Page 3 |
| 11 | Definitions of data elements, with references to Common Data Elements | Page 3 |
| 12 | De-identification methods | Not available |
| 13 | How missing data were handled | Page 2-3 |
| 14 | Definition of ground truth reference standard, in sufficient detail to allow replication | Page 3 |
| 15 | Rationale for choosing the reference standard (if alternatives exist) | Page 3-4 |
| 16 | Source of ground-truth annotations; qualifications and preparation of annotators | Page 3-4 |
| 17 | Annotation tools | Page 3 |
| 18 | Measurement of inter- and intrarater variability; methods to mitigate variability and/or resolve discrepancies | Page 3-4 |
| 19 | Intended sample size and how it was determined | Page 2-3 |
| 20 | How data were assigned to partitions; specify proportions | Page 3 |
| 21 | Level at which partitions are disjoint (e.g., image, study, patient, institution) | Page 2-3 |
| 22 | Detailed description of model, including inputs, outputs, all intermediate layers and connections | Supplemental documents |
| 23 | Software libraries, frameworks, and packages | Page 3-4 |
| 24 | Initialization of model parameters (e.g., randomization, transfer learning) | Not available |
| 25 | Details of training approach, including data augmentation, hyperparameters, number of models trained | Page 4-5 |
| 26 | Method of selecting the final model | Page 4-5 |
| 27 | Ensembling techniques, if applicable | Not applicable |
| 28 | Metrics of model performance | Page 8 |
| 29 | Statistical measures of significance and uncertainty (e.g., confidence intervals) | Page 5 |
| 30 | Robustness or sensitivity analysis | Page 6-7 |
| 31 | Methods for explainability or interpretability (e.g., saliency maps), and how they were validated | Page 4-5 |
| 32 | Validation or testing on external data | Not available |
|  |  | **RESULTS** |
| 33 | Flow of participants or cases, using a diagram to indicate inclusion and exclusion | Page 3 |
| 34 | Demographic and clinical characteristics of cases in each partition | Page 5 |
| 35 | Performance metrics for optimal model(s) on all data partitions | Page 6-8 |
| 36 | Estimates of diagnostic accuracy and their precision (such as 95% confidence intervals) | Page 6-8 |
| 37 | Failure analysis of incorrectly classified cases | Not applicable |
|  |  | **DISCUSSION** |
| 38 | Study limitations, including potential bias, statistical uncertainty, and generalizability | Page 9-10 |
| 39 | Implications for practice, including the intended use and/or clinical role | Page 11 |
|  |  | **OTHER INFORMATION** |
| 40 | Registration number and name of registry | Not applicable |
| 41 | Where the full study protocol can be accessed | Not available |
| 42 | Sources of funding and other support; role of funders | Title Page |

*From: Mongan J, Moy L, Kahn CE Jr. Checklist for Artificial Intelligence in Medical Imaging (CLAIM): a guide for authors and reviewers. Radiol Artif Intell 2020; 2(2):e200029.* [*https://doi.org/10.1148/ryai.2020200029*](https://doi.org/10.1148/ryai.2020200029)

Supplementary Table S2. Gadoxetic acid enhanced MRI scanning parameters.

| Phases | Flip  Angle | Field of  View(mm) | Repetition  time(ms) | Echo  time(ms) | Slice  thickness(mm) |
| --- | --- | --- | --- | --- | --- |
| Arterial phase | 13 | 400×400 | 3.42 | 1.25 | 2.5 |
| Portal phase | 13 | 400×400 | 3.42 | 1.25 | 2.5 |
| Equilibrium phase | 13 | 400×400 | 3.42 | 1.25 | 2.5 |
| Hepatobiliary phase | 30 | 400×400 | 3.42 | 1.25 | 2.5 |

Supplementary Material S1. Formula of the clinical model (Clin-model)

*Y* = -1.6592511 - 0.9150306*Platelet + 1.1616476*ALBI-score +1.5829295* ICG-R15

Supplementary Material S2. Formula for calculation of Rad-score based on Rad-model.

Rad-score = -0.00066*original_shape_Flatness+

0.04187*original_glcm_ClusterShade+

(-0.03329)*original_glszm_SizeZoneNonUniformityNormalized+

(-0.03241)*wavelet-LLH_firstorder_Maximum+

0.00042*wavelet-LLH_ngtdm_Busyness+

0.00762*wavelet-LHL_firstorder_Maximum+

(-0.003)*wavelet-LHL_glszm_LargeAreaHighGrayLevelEmphasis+

0.03397*wavelet-LHL_gldm_DependenceNonUniformityNormalized+

(-0.01423)*wavelet-LHL_gldm_DependenceVariance+

(-0.05515)*wavelet-LHH_glcm_InverseVariance+

(-0.00913)*wavelet-HHL_firstorder_Kurtosis+

0.00854*wavelet-HHL_glrlm_RunLengthNonUniformityNormalized+

(-0.00645)*wavelet-HHL_glrlm_RunVariance+

(-0.01442)*wavelet-HHL_glszm_SizeZoneNonUniformityNormalized+

(-0.03904)*wavelet-HHL_gldm_LargeDependenceEmphasis+

0.02409*wavelet-HHH_gldm_DependenceEntropy

Supplementary Figure S1. Rad-score in training and test cohorts.


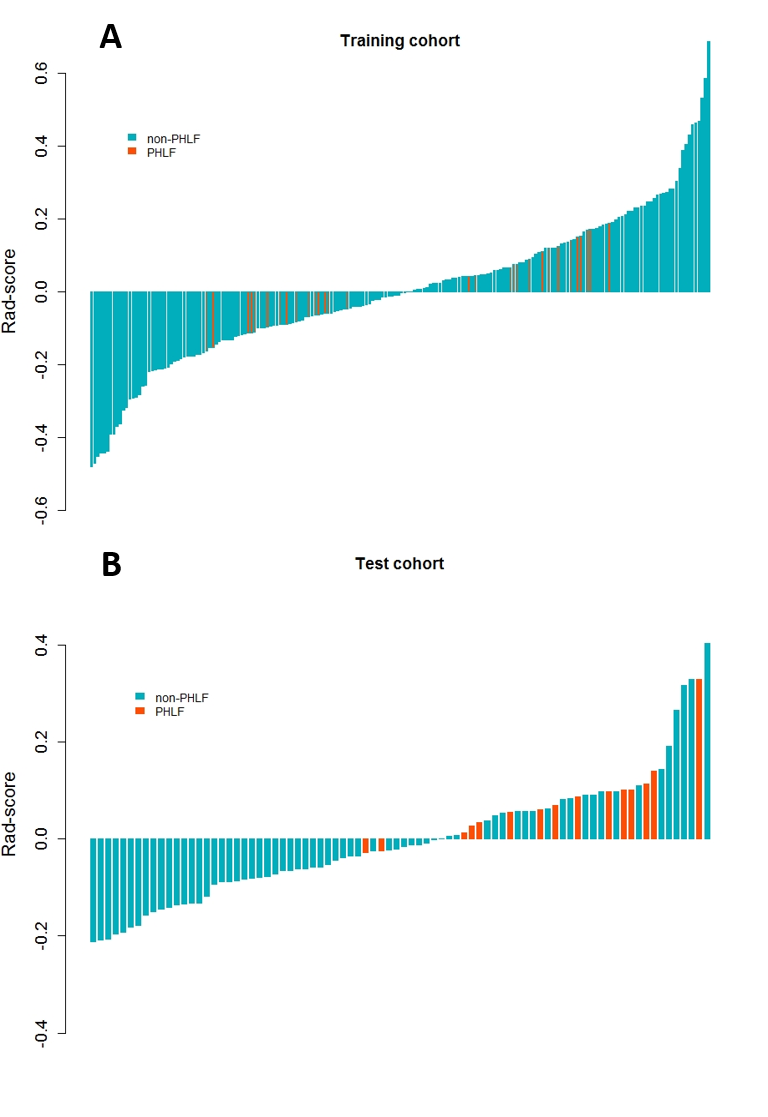


Supplementary Table S3. Comparison between the models with and without a variable of “resection extent”.

|  | | Clin-model | Clin-model+  resection extent | Combined model | Combined model+  resection extent |
| --- | --- | --- | --- | --- | --- |
| Training cohort | Cut-off value | 0.27 | 0.37 | 0.28 | 0.29 |
|  | AUC (95% CI) | 0.74 (0.65-0.83) | 0.73 (0.65-0.82) | 0.84 (0.77 - 0.90) | 0.84 (0.77-0.90) |
|  | Sensitivity | 0.70 | 0.54 | 0.78 | 0.76 |
|  | Specificity | 0.74 | 0.86 | 0.81 | 0.84 |
|  | PPV | 0.49 | 0.57 | 0.59 | 0.62 |
|  | NPV | 0.88 | 0.84 | 0.91 | 0.91 |
|  | Accuracy | 0.73 | 0.78 | 0.80 | 0.29 |
|  | ***p* (Delong test)** | **0.636** | | **0.984** | |
| Test cohort | AUC (95% CI) | 0.71 (0.57 – 0.84) | 0.72 (0.59-0.85) | 0.82 (0.72 - 0.91) | 0.81 (0.72-0.91) |
|  | Sensitivity | 0.87 | 0.87 | 0.93 | 0.87 |
|  | Specificity | 0.55 | 0.60 | 0.67 | 0.73 |
|  | PPV | 0.30 | 0.33 | 0.39 | 0.42 |
|  | NPV | 0.95 | 0.95 | 0.98 | 0.96 |
|  | Accuracy | 0.61 | 0.65 | 0.72 | 0.76 |
|  | ***p* (Delong test)** | **0.542** | | **0.988** | |
